# The ethics of mental health survey research in low- and middle- income countries

**DOI:** 10.1017/gmh.2016.6

**Published:** 2016-04-11

**Authors:** S. M. Murray, N. Kass, T. Mendelson, J. Bass

**Affiliations:** 1 Department of Mental Health, Johns Hopkins Bloomberg School of Public Health, 624 N. Broadway, Baltimore, MD 21205, USA; 2 Department of Health Policy and Management, Johns Hopkins Berman Institute of Bioethics, Baltimore, Maryland, USA; 3 Department of Mental Health, Johns Hopkins Bloomberg School of Public Health, Baltimore, Maryland, USA

**Keywords:** global health, mental health, research ethics

Neuropsychiatric conditions are a leading cause of disability globally (Whiteford *et al.* 2013), yet the gap between population need and the availability of evidence-based mental health services is wide even in high-income countries (Demyttenaere *et al.* 2004). Efforts to understand this gap have led to an expansion of global mental health (GMH) research, particularly in low-and middle-income countries (LMIC) where the availability of treatment services is even more severely lacking (Demyttenaere *et al.* 2004; Patel, 2007), including administration of surveys that are not associated with an intervention or service (Alonso *et al*. 2013). Such surveys can provide data on unmet needs, be used to advocate for resources, and elucidate etiological processes; they also, however, pose ethical challenges. Guided by the ethical principles of beneficence, autonomy, and justice (National Commission for the Protection of Human Subjects of Biomedical and Behavioral Research, 1978; Council for International Organizations of Medical Sciences, 2002), we discuss these challenges and present steps GMH researchers can take to further incorporate these ethical principles into practice during the design of new studies in LMIC contexts.

## Ethical challenges in GMH survey research

Beneficence compels researchers to minimize harms and ensure that the benefits of research outweigh risks (National Commission for the Protection of Human Subjects of Biomedical and Behavioral Research, 1978). Survey participants generally receive little if any direct benefit for their involvement; rather, the benefit of GMH surveys is typically the potential social good of new knowledge about the nature and burden of poor mental health. This can be problematic for three reasons. First, ensuring that a societal benefit results from a GMH survey is not always straightforward. If the knowledge produced is neither novel nor useful, then the study is not producing benefit no matter how low the risk. The challenge lies in determining what is novel and useful and for whom. For example, in disaster and conflict settings, numerous studies have already demonstrated that many people experience depression, anxiety, and post-traumatic stress symptoms (Steel *et al.* 2009). A survey in a new conflict setting to identify the prevalence of elevated symptoms would produce novel knowledge only if a strong scientific rationale existed for thinking that previous research (due to contextual factors or methodological flaws) is not applicable to the new setting.

Second, when benefits are societal and long-term, researchers have a responsibility to communicate to potential participants that the study is *not* designed to help them in the short-term. In LMIC settings, this often poses challenges for obtaining informed consent, a cornerstone of ethical research that is based on upholding the principle of autonomy. If the information provided on the aim and content of the survey is flawed (e.g. recognizable local terminology for mental health disorders and symptoms are not used), potential participants cannot provide adequately informed consent (Summerfield, 2008). Further, especially if a community has limited familiarity with research, asking personal questions about mental health could engender the idea that services are forthcoming or that the researcher is there to assist despite stating in the informed consent process that the survey has no direct benefit. For instance, participants in psychiatric research trials in high-resource settings have displayed a ‘therapeutic misconception’; despite explanation of randomization and a treatment as usual or placebo condition, many participants believed their treatment would be determined by their medical needs and that they would directly benefit from the study (Appelbaum *et al*. 1982). While trial research can present substantially different opportunities for benefit (e.g. control group participants may receive treatment after trial completion), this demonstrates that individuals may persist in believing that help will be provided once researchers become aware of their needs regardless of what is presented in a consent form.

Finally, that benefits are uncertain also raises questions regarding justice in GMH research. To promote distributive justice, populations involved in research must receive ‘fair benefits’ proportional to the risk or burden posed by the study (Participants in the 2001 Conference on Ethical Aspects of Research in Developing Countries, 2002). This is of particular concern when studying vulnerable populations for whom risks may be higher. Persons with mental health needs in LMIC may be vulnerable due to multiple interacting factors including displacement due to conflict or natural disaster, belonging to a group with relatively low status, experiencing discrimination due to their illness, or having no access to mental health resources or care (Council for International Organizations of Medical Sciences, 2002; Siriwardhana, 2015). Although surveys are not interventions, it is incorrect to assume that they cannot impact participants in both positive and negative ways, including by posing risks. An example of such a risk noted above is mistaken and disappointed expectations of research participants.

Risk is contextual and can vary depending on a complex interplay between environmental factors and participant characteristics, including gender, age, marital status, sexual identity, ethnicity, and income (Ruiz-Casares, 2014). Assessing risk can therefore be a challenge for an investigator working with populations that differ markedly in culture, socioeconomic status, or familiarity with research, whether or not the population lives in the investigator's home country. For example, diagnostic instruments that rely on definitions of disorder created in high-resource settings without consideration for local conceptions of mental health risk committing category fallacy and reifying a problem that is not recognized or experienced in the same way locally (Kleinman, 1977). Additionally, while stigma associated with mental health conditions is a global problem (Drew *et al.* 2011), stigma can vary in nature and type. GMH researchers unfamiliar with a study population could inadvertently identify those suffering with a stigmatized condition to their community putting them at increased risk. This is of particular concern when strategies such as snowball sampling are used; while useful for engaging hard to reach participants, these methods can compromise confidentiality if not carefully implemented (Jacobsen & Landau, 2003). Community-level harms can also result if groups are identified through research as being associated with a stigmatized behavior or experience (American Academy of Pediatrics, Committee on Native American Child Health and Committee on Community Health Services, 2004).

## Strategies for researchers

Recognizing the importance of these challenges, researchers can employ several strategies to increase the ethical acceptability of GMH surveys (Table 1). A thorough literature review is a necessary but insufficient step toward ensuring that a survey provides a societal benefit through the production of new knowledge. Such a review may also help researchers identify data sets whereby novel questions can be answered through secondary analysis without burdening a new set of participants. Determining the sufficiency of the state of accumulated knowledge on a given topic is a complex task. Therefore, in the following we suggest additional strategies that researchers can employ to increase the likelihood that the knowledge produced by a survey will generate future benefits that could aid in balancing risks or concerns a survey might entail.

**Table 1.** Summary of ethical challenges and potential solutions for global mental health survey research

Ethical challenge	Guiding principle(s)	Questions for investigators and IRBs to ask of new research	Questions for communities to ask of new research and researchers	Potential solutions
Ensuring research findings are useful and novel	Beneficence	• Will the research produce unique findings that can impact mental health policy or services? • Does answering this question necessitate burdening new participants?	• How likely is it that the research will result in changes for the communities from which participants are drawn?	• Ensure a thorough literature review has been done to identify studies of mental health in similar populations. Only proceed if no substantial research exists on the topic that is generalizable to the population of interest. • Look for other data sets already in existence that could be used to answer new research questions before beginning a new study.
Including and protecting vulnerable participants	Justice and beneficence	• Has risk and vulnerability been appropriately assessed and addressed in the study context? • Is the likelihood of direct benefit proportional to the degree of participant vulnerability?	• Do the researchers have an understanding of the problems of participants and/or a plan for receiving ongoing feedback? • Do the researchers have a plan for sharing findings with the participants and their communities? Who will ‘own’ the results?	• Use community-based advisory boards and participant input to ensure protection of individuals, adequate assessment of risk, a full understanding of vulnerability, and threats to identifiable communities. • Embed surveys into ongoing mental health service provision. • If not possible, find a government group or community-based organization interested in providing mental health services with whom to partner. Build capacity and share ownership of results. • Together use the results to advocate for funding for an intervention or for policy/legal reform. • If no organization is interested, conduct participatory research so participants can provide input and put findings to use locally.
Accurately communicating benefits and managing expectations of research	Autonomy	• Will the community's expectations of research be fairly managed? • Will potential participants recognize this mental health issue and be able to assess risks and benefits of the research?	• Do the researchers know what community members and participants expect to happen as a result of the research? • Will participants and their communities understand the purpose and nature of the study as explained by the researchers?	• Solicit input from community and participant advisory boards on research expectations and how to clearly communicate the purpose, limits, and potential benefits of the research to stakeholders and participants. • Conduct initial formative exploratory research to elicit local understandings, terminology, and relevance of the problem of interest. Use findings together with knowledge of context, adherence to ethical guidelines, and ethical review structures to create an appropriate informed consent procedure and to accurately assess and explain potential benefits.

Working with community-based partners or creating a participant advisory board early in the research process can provide investigators with valuable local insight. Investigators can learn about the utility and importance of mental health concepts to local communities, hear how local communities frame their symptoms and concerns, understand expectations that community members may have of researchers who ask about mental health, and assess vulnerability of the target population (Varmus & Satcher, 1997; Beyrer & Kass, 2002; Ruiz-Casares, 2014). A formative planning phase that includes discussions with local service providers, community organizations, and community members can garner local views on these topics and enable researchers to gain insight on local attitudes and stigma associated with mental health problems (Allden *et al.* 2009).

Risks of a GMH survey can be minimized through sampling decisions, specifically by selecting the least vulnerable sample to meet the needs of the study aims. For example, in many settings poverty may be widespread and resources for mental health extremely constrained, making much of the population highly vulnerable as they might lack any alternative avenues to receive care (Council for International Organizations of Medical Sciences, 2002) and as discussed may perceive research as a gateway to services. However, there may be some groups that are less vulnerable, such as adults from the general population as opposed to a specific group like torture or sexual violence survivors. Such individuals may experience stigma and relatively lower social status as a result, can impair one’s ability to safeguard his or her own interests (Council for International Organizations of Medical Sciences, 2002). If the aims allow for selection of a broader group of participants, this could minimize the focus on more highly vulnerable groups. Even so, a population should not be omitted from GMH research simply due to vulnerability (Emanuel *et al*. 2000; Beyrer & Kass, 2002), and blanket characterization of a heterogeneous group as vulnerable could prevent individuals from exercising agency in the choice to participate. The important point for investigators to remember is that research among vulnerable groups is only justifiable when it is realistically plausible that an intervention would become accessible to them (Beyrer & Kass, 2002) or that it would lead to policies that improve their access to services.

Determining how likely participants will be able to access services is challenging and may feasibly occur only in the longer term. However, researchers can improve the benefit–risk ratio for participants by employing strategies to tie research to service provision. If the knowledge produced by a survey is irrelevant (e.g. due to the use of diagnostic instruments that are not culturally adapted) or unimportant to participants, then the research will have little future potential to inform acceptable and needed services for that community. Ideally, survey research would be embedded into ongoing mental health programming. If partnering with local mental health service providers is not possible–and sadly in LMIC contexts mental health services will often not exist–investigators can partner with governments, international NGOs, or community-based organizations interested in providing mental health services and can help them develop appropriate programs based on survey results. Through sustainable partnership, researchers can build the capacity of local organizations to use results to advocate for themselves for funding, policy reform, or new laws to improve mental health locally. If no potential organizational partner exists, groups of community members formed through participatory research or existing community social structures could serve the same role (see Vreeman *et al*. 2012). Solely publishing survey results in a scientific journal may not be sufficient to generate policy change or services in the community where research takes place, but through partnerships, securing resources to provide benefits to participants becomes more likely. For example, using participatory methods, Afifi *et al.* (2011) developed and successfully pursued funding for an intervention following a needs assessment with refugee youth.

At an absolute minimum, GMH researchers must act to keep participants safe during survey research and take steps to monitor any negative ramifications of the survey on individuals’ safety or well-being. Murray *et al.* (2014) present some options for safety planning when formal mental health services are not available in LMIC. Topics such as what questions to ask if participants express suicidal ideation, how to elicit community resources, and strategies for involving friends and family in safety monitoring will be relevant for GMH survey researchers. For instance, if a participant indicates in a response to a depression questionnaire that they have had thoughts of death or ending their lives, this necessitates an evaluation from research staff of the level of risk (e.g. does the person have a plan, access to means, or do they have a history of prior attempts) (Murray *et al.* 2014). Responding yes to these questions would indicate high-risk and trigger further protective action. Partnerships and discussions with local organizations and advisory broads can help researchers understand the mental health landscape for referral of high-risk cases identified during survey implementation, including available treatment resources and the quality of available care (i.e. to avoid legitimizing ineffective or abusive care). Partnerships can also provide information needed by researchers to meet the obligation of providing appropriate safety planning when cases of serious mental distress accompanied by suicidal ideation or abuse are identified and there are no local mental health resources.

The extent to which investigators are responsible for the mental health treatment of such participants, both in terms of time and resources, will vary. Investigators should consider the level of risk the individual undertook in participating, their dependency on the researcher to receive any form of care, and the depth of the relationship between the researcher and participant including the level of trust engendered when individuals provided sensitive details about their mental health (Belsky & Richardson, 2004). When designing a survey, researchers should also consider the sensitivity of the questions being asked and whether the questions are essential. For instance, if doing a survey on depression, it may not be important to assess history of trauma exposure unless directly applicable to the research question. If researchers simply are trying to assess the overall prevalence of trauma exposures, using a ‘neighborhood method’ may minimize the burden on participants to reveal sensitive or stigmatizing information about themselves and provide some level of anonymity (Potts *et al.* 2011).

## Conclusions

Although we have focused on GMH research conducted in LMIC, many issues discussed apply equally to mental health research in high-income countries despite the potential for greater access to treatment resources in these contexts. Guidance on ethically conducting GMH research, including intervention research, is increasingly available (Ellis *et al.* 2007; Allden *et al.* 2009; Siriwardhana *et al.* 2013; Ruiz-Casares, 2014; Chiumento *et al.* 2015). This is essential as implementing and evaluating interventions presents additional ethical challenges. However, given the complexities of GMH research, careful and ongoing consideration is warranted. For example, while partnership with local organizations and groups is a promising solution, implementing this can be fraught with challenges as communities are not homogenous and individuals within the community may have different motivations related to engaging with research. Researchers may face challenges of hierarchy in the relationships they have with community advisors as well as between community members themselves (Puffer *et al*, 2013). One promising mechanism that has been suggested for promoting greater ethical consideration in GMH is a post-research audit (Srirwardhana, 2015). Audits encourage ongoing monitoring of negative ramifications of study participation, facilitate the sharing of successful strategies for employing ethical principles in practice, and identify gaps between available and needed guidance.

The Working Group on Mental Health and Psychosocial Support convened at Harvard's Humanitarian Action Summit in 2009 argued that mental health research in disaster and conflict settings must directly benefit the community under study in order to be ethical (Allden *et al.* 2009). As a general guide in LMIC settings more broadly, integrating survey research with services becomes increasingly critical at higher levels of participant vulnerability. If researchers demonstrate that their survey will address a meaningful and socially valuable gap in knowledge, the strategies described here for minimizing risk and raising the likelihood of future direct benefit may lead to a more ethically acceptable study. Since the determination of whether or not research can responsibly proceed depends on context, partnering with local groups is essential. To ensure ethical treatment, every researcher must address the challenges presented here together with ethical review boards, communities, and participants from study inception to dissemination.
